# IL-1β-Triggered Long Non-coding RNA CHRF Induces Non-Small Cell Lung Cancer by Modulating the microRNA-489/Myd88 Axis

**DOI:** 10.7150/jca.63256

**Published:** 2022-05-16

**Authors:** Yamei Zhang, Yabo Zhang, Qianglin Zeng, Ci Li, Hui Zhou, Junying Liu, Zheng Shi, Li Ma

**Affiliations:** 1 Clinical Genetics Laboratory, Affiliated Hospital & Clinical Medical College of Chengdu University, Chengdu 610081, P.R. China.; 2 Department of Respiratory and Critical Care Medicine, Affiliated Hospital & Clinical Medical College of Chengdu University, Chengdu 610081, P.R. China.; 3 Department of Pathology, Affiliated Hospital & Clinical Medical College of Chengdu University, Chengdu 610081, P.R. China.; 4 Institute of Blood Transfusion, Chinese Academy of Medical Sciences, Chengdu 610052, P.R. China.

**Keywords:** Long noncoding RNA cardiac hypertrophy-related factor, microRNA-489, Myeloid differentiation factor 88, Interleukin-1β, Non-small cell lung cancer, Proliferation, Migration, Invasion, Apoptosis

## Abstract

**Background:** Long noncoding RNAs (LncRNAs) possess crucial roles in carcinogenesis. The current study aims to evaluate the effects of interleukin-1β (IL-1β)-mediated lncRNA cardiac hypertrophy-related factor (CHRF)/microRNA-489 (miR-489)/myeloid differentiation factor 88 (Myd88) on non-small-cell lung cancer (NSCLC).

**Methods:** Initially, the expression of IL-1β and lncRNA CHRF in NSCLC cells and tissues was determined, respectively. H460 cell line with highest lncRNA CHRF expression was selected for *in vitro* experimentations. Afterward, the interaction among lncRNA CHRF, miR-489, and Myd88 was verified with their significance in cell functions and tumorigenicity and lung metastasis analyzed following.

**Results:** IL-1β and lncRNA CHRF was remarkably upregulated in NSCLC. IL-1β was able to elevate lncRNA CHRF expression. Additionally, lncRNA CHRF targeted miR-489 and miR-489 targeted Myd88. Moreover, functional assay results suggested that under IL-1β treatment, lncRNA CHRF induced NSCLC cell malignant properties and tumorigenicity and lung metastasis through modulation of miR-489/Myd88 axis.

**Conclusion:** Taken together, our findings revealed that IL-1β-induced elevation of lncRNA CHRF aggravated NSCLC through modulation of miR-489/Myd88 axis, which provides a novel direction for NSCLC therapy development.

## Introduction

Lung cancer is a leading cause of cancer-related deaths worldwide and claims more lives each year than any other cancer do. Moreover, approximately 80% of mortality caused by lung cancer can be ascribed to non-small cell lung cancer (NSCLC), representing the most common type of lung cancer. Unfortunately, it can only be diagnosed at its advanced stages 1, 2. Being aggressive cancer, NSCLC is characterized by its rapid progression and relatively low survival rates 3. At present, the available treatment options for patients suffering from metastatic NSCLC include chemotherapy, targeted therapy, and immunotherapy 4. Recent researches have made tremendous progress regarding NSCLC therapy, however, the 60 month-survival rate remained low 5. The high failure rate in the current treatments of NSCLC and lack of effective therapy remain to be a significant hindrance in the management of this disease, reflecting an urgent requirement for exploring the novel molecular biomarkers and therapeutic targets for NSCLC 6.

Long noncoding RNAs (lncRNAs) exert diverse biological functions, such as gene transcription modulation, regulation of apoptosis, and invasion, as well as microRNA (miRNA) host genes 7. Wu *et al.* have found that silica-induced lung fibrosis can be inhibited by miR-489 through targeting the myeloid differentiation factor 88 (Myd88) and Smad3, however, miR-489 has been demonstrated to be negatively regulated by lncRNA cardiac hypertrophy-related factor (CHRF) 8. Additionally, the tumor-suppressive role of miR-489 has been reported in gastric cancer and pancreatic carcinoma 9, 10. Myd88 is known as a well-known important adapter protein, mediating the toll-like receptor and the downstream components to activate the related signaling pathways 11. Wang *et al.* have proved the regulation of Myd88 by miR-489 in cardiac hypertrophy while lncRNA CHRF can directly bind to miR-489 and further regulate the Myd88 expression 12. In addition, interleukin-1β (IL-1β), a pro-inflammatory cytokine, is a well-established key mediator in infection, inflammation, and immunity in mammals 13. Moreover, IL-1β is capable of modulating miR-101 expression in lung tumorigenesis promoted by inflammation 14*.* Considering these aspects, we anticipated that the lncRNA CHRF/miR-489/Myd88 axis might be a promising biomarker for patients with NSCLC. Hence, we attempted to unveil the function and inter-relationships among IL-1β, lncRNA CHRF, miR-489, and Myd88 in the development and metastasis of NSCLC.

## Materials and methods

### Ethics Statement

Under approval of the Ethics Committee of Affiliated Hospital of Chengdu University, this study was implemented with written informed consent provided by each participant or their relatives before enrollment. Animal experiments were started referring to the Guide for the Care and Use of Laboratory Animals promulgated by the National Institutes of health.

### Study Subjects

NSCLC and adjacent normal tissues were gained from 62 patients diagnosed with NSCLC by pathology and underwent surgical treatment in Affiliated Hospital & Clinical Medical College of Chengdu University, from May 2017 to December 2019. These patients did not receive any local or systemic therapy before surgery. Patients were followed up by telephone one time every two months for consecutive 5 years after surgery. The disease-free survival (DFS) of patients was analyzed. The specimens were evaluated by histopathology with detailed clinical data shown in Supplementary Table 1. All specimens were cryopreserved in liquid nitrogen immediately after being taken out and then stored at -80 ºC.

Patients were enrolled if 1) they were diagnosed as NSCLC with clinical stage clearly identified before surgery according to lung cancer staging criteria in American Joint Committee on Cancer Eighth Edition; 2) they underwent standardized treatment based on National Comprehensive Cancer Network Clinical Practice Guidelines for lung cancer; and 3) their age ranged between 18 and 70 years old.

Patients were excluded if 1) they suffered from metastatic lung cancer or other types of malignancies; 2) they had serious chronic disease in the heart, brain, liver or kidney; 3) their clinical data were incomplete or they were lost to follow-up; or 4) they died due to other diseases during follow-up.

### Cell Culture

One normal lung epithelial cell line (16HBE) (Procell, Wuhan, Hubei, China) and 3 NSCLC cell lines (H460, H1299, and A549) (ATCC, Manassas, VA) were cultured in Royal Park Memorial Institute (RPMI) 1640 culture medium appended to 10% newborn serum, 100 U/mL penicillin and 100 U/mL streptomycin at 37 ºC with 5% CO_2_. The culture medium was renewed every two days. The cells were sub-cultured upon reaching 80% - 90% confluence and logarithmically growing cells were chosen for subsequent experimentations.

### Enzyme-linked immunosorbent assay (ELISA)

All cells were cultured in an incubator at 37 ºC with 5% CO_2_ for 2 days. The collected supernatant of cell culture was centrifuged at 1000 ×g for 20 minutes to harvest the supernatant. The tissue samples were rinsed with pre-cold phosphate-buffered saline (PBS) to remove the residual blood. After weighing, the tissues were cut into pieces and fully ground on ice in a glass homogenizer with PBS at a volume ratio of 1:9. The homogenate was broke into pieces by ultrasonic sound to further lyse tissues and cells. The supernatant was collected through centrifugation at 5000 ×g for 10 minutes. The content of IL-1β in NSCLC tissues, adjacent normal tissues and cells was tested by the IL-1β ELISA kit (#DLB50, R&D Systems, Minneapolis, MN).

### Plasmid Construction and Transfection

The sequences of lncRNA CHRF and miR-489 were retrieved from the National Center for Biotechnology Information. The sh-lncRNA CHRF, sh-lncRNA CHRF-negative control (NC), miR-489 mimic, miR-489 mimic-NC, miR-489 inhibitor, and miR-489 inhibitor-NC plasmids constructed by Sangon Biotech Co., Ltd. (Shanghai, China) were chosen for cell transfection utilizing Lipofectamine 2000 (Invitrogen, Carlsbad, CA). Three different types of plasmids were evaluated and the most precise and effective plasmid was selected to avoid any off-target effect. IL-1β was added accordingly with the final concentration of 10 ng/mL 15-17. To screen out stably transfected cells, 48 hours after transfection the cells were treated with G418 medium (1000-2000 μg/mL) for 4 weeks and the culture medium was renewed every 3 to 5 days.

### Reverse transcription-quantitative polymerase chain reaction (RT-qPCR)

Trizol reagent (Takara Inc., Dalian, Liaoning, China) was employed to extract total RNA, whose purity and concentration were tested by NanoDrop ND-1000 spectrophotometer (Nanodrop Technologies, Wilmington, DE, USA). miRNA was reversely transcribed into cDNA using MiRcute miRNA first chain cDNA synthesis kit (Tiangen Biotech, Beijing, China) and mRNA was reversely transcribed using Primer-Script TM one-step RT-PCR kit (Takara, Shiga, Japan). The Quanti-Tect SYBR Green PCR Kit and the SYBR Premix Ex Taq II Kit (Takara) were applied for performing RT-qPCR with U6 and glyceraldehyde-3-phosphate dehydrogenase (GAPDH) as normalizers. The primers are shown in Supplementary Table 2. All RT-qPCR experimentations were implemented on the ABI7500 qPCR instrument (Applied Biosystems, Foster City, CA). The relative expression of each gene was determined by the 2^-ΔΔCt^ method.

### Western blot analysis

The total protein was extracted with the Radio Immunoprecipitation Assay kit (R0010, Beijing Solabio Life Sciences Co., Ltd., Beijing, China), and the protein concentration was determined using the bicinchoninic acid protein assay kit (GBCBIO Technologies Co., Ltd., Guangzhou, China). A total of 40 µg of each sample was loaded, electrophoresed with 6%, 10%, and 15% sodium dodecyl sulphate-Xpolyacrylamide gel electrophoresis, and transferred to the polyvinylidene fluoride membrane (Millipore, MA). Following blockage using Tris-buffered saline Tween solution with 5% bovine serum albumin at ambient temperature, the membrane was reacted with diluted primary antibody to Myd88 (ab133739, 1:2000, Abcam Inc., Cambridge, UK) overnight at 4 ºC as well as with secondary antibody of goat anti-rabbit immunoglobulin G (IgG, ab97051, 1 : 2000, Abcam) at room temperature for 2 hours. Then, the Western blots were developed by electrochemiluminescence and imaged on Image Quant LAS 4000C (GE Healthcare, Little Chalfont, Buckinghamshire, UK).

### RNA Fluorescence *In situ* Hybridization (FISH) Assay

FISH technology was used to identify the subcellular localization of lncRNA CHRF according to the instructions of the FISH detection kit (Boster Biological Technology Ltd., Wuhan, Hubei, China) 18. Five different fields of vision were selected and photographed under fluorescent confocal microscopy (Olympus, Tokyo, Japan).

### Dual-Luciferase Reporter Gene Assay

miR-489 was predicted as a regulatory miRNA of lncRNA CHRF and Myd88 as a target gene of miR-489 by the bioinformatics website, which was further confirmed by the dual-luciferase reporter gene assay. Shortly, the lncRNA CHRF-wild type (WT)-Luc and lncRNA CHRF-mutant (MUT)-Luc plasmids were constructed and Myd88-WT-Luc and Myd88-XMUT-Luc were bought from GeneChem (Shanghai, China). 293T cells (ATCC) were co-transfected with the lncRNA CHRF-WT-Luc, lncRNA CHRF-MUT-XLuc, Myd88-WT-Luc, or Myd88-MUT-Luc with miR-489 mimic and miR-489 mimic-NC using the Lipofectamine^TM^ 2000 (Invitrogen). After 24 h transfection, the luciferase activity was evaluated at 560 nm wavelength employing the Dual-Luciferase Reporter Assay Kit (Promega, Madison, WI) and the microplate reader (MK3, Thermo Fisher Scientific Inc., Waltham, MA).

### RNA Pull-Down Assay

The AmpliScribe T7-Flash Biotin-RNA Transcription Kit (Epicentre, Madison, WI) was adopted to transcribe RNA which was then purified by the RNeasy Plus Mini Kit (Qiagen, Hilden, Germany) and DNase I (Qiagen) and biotin-labeled using the Biotin RNA Labeling Mix Kit (Ambio Life, San Antonio, Texas, USA). RNA structure buffer containing 10 mM Tris (pH = 7.0), 0.1 M KCl and 10 mM MgCl_2_ was added to the biotin-labeled RNA to form a suitable two-grade structure and incubated for 2 minutes at the heat shock temperature of 90 °C, followed by the incubation on ice for 30 minutes and then a further incubation at ambient temperature for 20 minutes. RNA was mixed with the extracted total protein of cells in each group and incubated at ambient temperature for 1 hour. Thereafter, the streptavidin labeled magnetic beads (Streptavidin Mag Sepharose, GE Healthcare) were added and the mixture was incubated for 1 hour at ambient temperature. Afterward, the cells were washed with ddH_2_O to obtain the final RNA-pull down complex which was gained for subsequent RT-qPCR.

### RNA-Binding Protein Immunoprecipitation (RIP) Assay

The RIP assay was performed employing the Magna RIP^TM^ RNA-Binding Protein Immunoprecipitation Kit (Millipore). The cells were lysed with RIP lysis buffer and reacted with RIP buffer with magnetic beads containing anti-human Argonaute 2 (Ago2) antibody or IgG protein (NC) at ambient temperature for 1 hour followed by 5 times washing with RIP buffer. After that, the washing solution was collected for RT-qPCR detection.

### Cell Counting Kit-8 (CCK-8) Assay

After 48 hours of transfection, the logarithmically growing cells were made into cell suspension (1 × 10^4^ cells/mL) by RPMI-1640 with 10% fetal bovine serum (FBS) and then transferred to 96-well plates (100 μL/well) for incubation in a 5% CO_2_ incubator at 37 ºC. Eight duplicated wells were constructed for each group. The culture plates were taken out at 24, 48 and 72 hours, respectively, while 10 µL of CCK-8 solution (Sigma-Aldrich, St. Louis, MO) was added for another 2-hour of incubation. The optical density was read at 450 nm wavelength using a microplate reader (MK3, Thermo Fisher Scientific Inc., Waltham, MA).

### Flow Cytometry

The cells were collected 48 hours after transfection and detached by 0.25% trypsin with the cell number adjusted to 1 × 10^6^ cells/mL. Following centrifugation at 402 ×g for 10 minutes, the cells were fixed by 70% pre-cold ethanol and preserved overnight at 4 ºC. Thereafter, the cells were washed 2 times with PBS and 100 μL of suspension was taken and incubated with 50 μg of propidium iodide (PI) staining solution containing RNAase (40710ES03, Shanghai Qcbio Science & Technology Co., Ltd., Shanghai, China) for 30 minutes (dark condition).

For cell apoptosis analysis, the Annexin V-FITC/PI double staining was performed. After culturing, the cells were washed 2 times with PBS, resuspended in 200 μL of binding buffer solution, and reacted with 10 μL of Annexin V-FITC (ab14085, Abcam) and 5 μL of PI staining solution at ambient temperature for 15 minutes (dark condition). After that, the cells were reacted with 300 μL of binding buffer solution. Cell cycle and apoptosis were tested both at the 488 nm excitation wavelength employing the flow cytometer (BD, FL, NJ).

### Transwell Assay

The Transwell chamber (Costar, High Wycombe, UK) with a diameter of 8-mm was adopted to evaluate cell migration and those pre-coated with 50 mg/L Matrigal (Millipore) were chosen for cell invasion capability evaluation 19. The cell migration or invasion was determined at 570 nm wavelength by a microplate reader (MK3, Thermo Fisher Scientific Inc., Waltham, MA, USA).

### Tumor Xenograft in Nude Mice

The 5-week-old female BALB/c nude mice (Dashuo Laboratory Animal Technology Co. Chengdu, China) were selected for experiments with 5 mice in each group. The mice were granted with ordinary food (water freely) and housed in well-ventilated cages at ambient temperature with a humidity of 50-60% and a 12-hour light/dark cycle.

Following 24 hours of transfection, the cells were detached, resuspended in serum-free RPMI 1640 medium, and counted. The cell suspension (1.5 × 10^6^ cells in 0.1 mL serum-free RPMI-1640) was mixed with 0.1 mL of extracellular matrix gel and then subcutaneously injected into the back of nude mice. The tumor formation was observed every 3 days after the injection followed by the measurement of the tumor volume. The mice were euthanized 4 weeks after the tumor was formed and the weight and volume of the tumor specimens were measured.

### Lung Metastasis of Tumor in Nude Mice

After 24 hours of transfection, the cells from each group were detached, washed two times with PBS and resuspended in serum-free RPMI 1640 medium to prepare the cell concentration into 2 × 10^6^ cells/mL. The injection of 1 mL cell into each 5-week-old female BALB/c nude mouse was carried out via tail vein. Mice were euthanized 30 days after injection. The lung tissues were taken and fixed by 10% neutral phosphate-buffered formalin to prepare lung specimens and the number of metastatic tumor corpuscles was counted.

### Statistical Analysis

Statistical analysis was conducted using the Statistical Package for the Social Sciences (SPSS) 21.0 (IBM Corp., Armonk, NY, USA). Measurement data were described as the mean ± standard deviation. Data between NSCLC tissues and adjacent normal tissues were compared by paired *t*-test. Comparison between the other two groups was analyzed using unpaired *t*-test and comparison among multiple groups were assessed using one-way analysis of variance (ANOVA) in combination with Tukey's post hoc test whereas the data at different time points were analyzed by the Bonferroni-corrected repeated measures ANOVA. Survival analysis was performed by means of Kaplan-Meier method and log-rank test. *p* < 0.05 and *p* < 0.001 were considered statistically significant.

## Results

### IL-1β and lncRNA CHRF were Highly Expressed in NSCLC Cells and Tissues

Of note, lncRNA CHRF has been tested to be highly expressed in cervical cancer, gastric cancer and prostate cancer in contribution to malignant cell proliferation, migration and drug resistance 20-22. Recently, IL-1β as a classical pro-inflammatory factor possesses promoting effect on proliferation and metastasis of NSCLC cells 23. Also, high expression of IL-1β and lncRNA CHRF has been detected in mice with sepsis while knockdown of lncRNA CHRF reduces inflammatory reaction 24.

**Figure 1:**
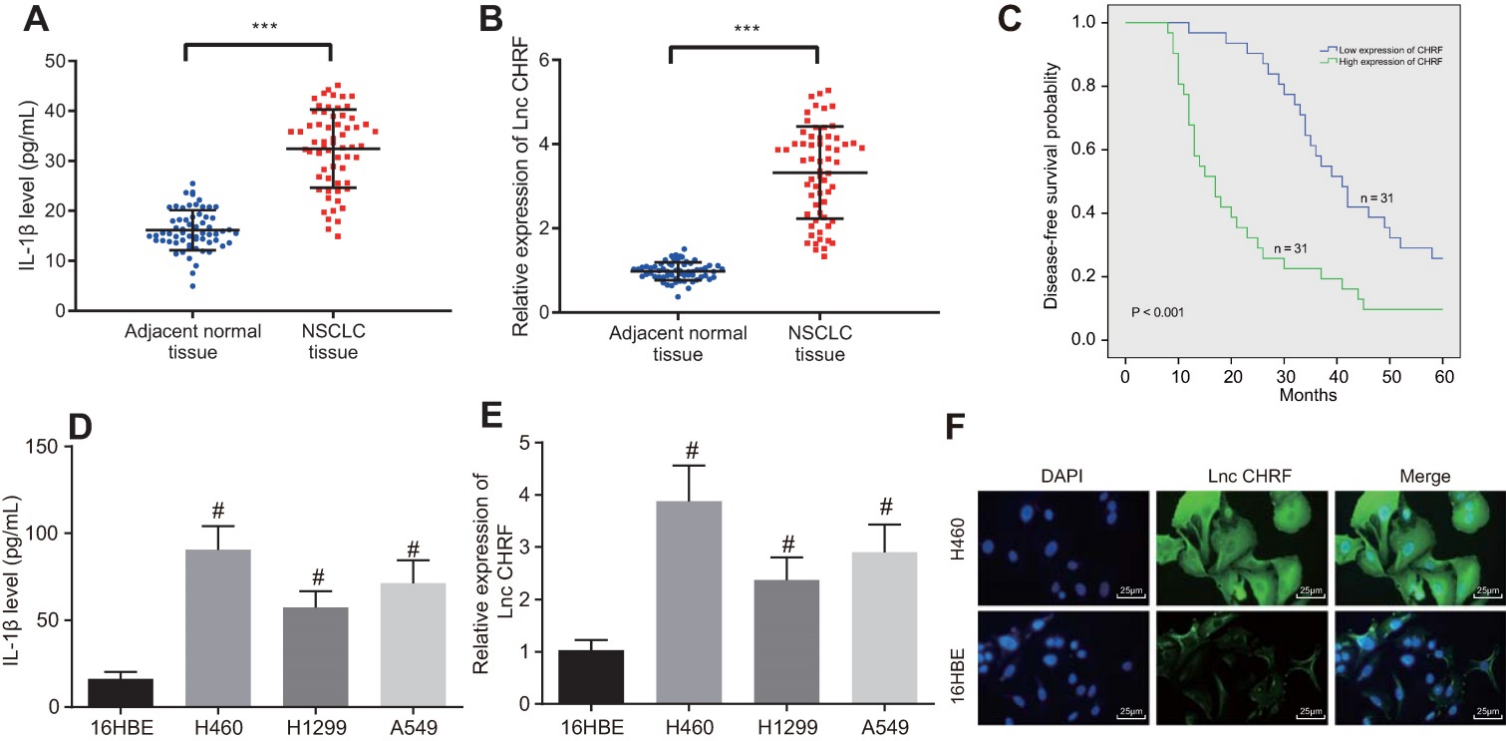
** IL-1β and lncRNA CHRF are highly expressed in NSCLC cells and tissues. A,** IL-1β expression in NSCLC tissues and adjacent normal tissues (n = 62) determined by ELISA. **B,** LncRNA CHRF expression in NSCLC tissues adjacent normal tissues (n = 62) determined by RT-qPCR. **C,** Relationship between the level of lncRNA CHRF and the prognosis of patients with NSCLC. **D,** IL-1β content in cell supernatant measured by ELISA. E, LncRNA CHRF in normal lung epithelial cell line (16HBE) and NSCLC and cell lines, including H460, H1299, A549 determined by RT-qPCR. F, Distribution of lncRNA CHRF in NSCLC cells detected by RNA-FISH assay, scale bar: 25 µm. *** *p* < 0.001 *vs.* adjacent normal tissues; # *p* < 0.05 *vs.* 16HBE cell line. The experiment was repeated 3 times independently. Data of NSCLC tissues and adjacent normal tissues were compared by paired* t* test, and the data of different cell lines were compared using one-way ANOVA, followed by Tukey's post hoc test.

Here, we attempted to further investigate the functional roles of IL-1β and lncRNA CHRF in NSCLC. As reflected by ELISA, the expression of IL-1β was increased in the NSCLC tissues (Figure 1A). RT-qPCR showed an increase of lncRNA CHRF in NSCLC tissues (Figure 1B). Furthermore, an inverse relation between lncRNA CHRF expression and DFS of patients with NSCLC was identified (Figure 1C).

Then, lncRNA CHRF expression in NSCLC cells and IL-1β content in the supernatant were measured. As detected by ELISA and RT-qPCR, compared with normal lung epithelial cell line 16HEB, the expression of IL-1β and lncRNA CHRF was higher in the three NSCLC cell lines H460, H1299, and A549, of which the H460 cell line with the highest expression of IL-1β and lncRNA CHRF was selected for subsequent experiments (Figure 1D, E). Additionally, the results of RNA-FISH assay exhibited that lncRNA CHRF was mainly located in the cytoplasm of H460 cell line (Figure 1F).

### LncRNA CHRF Promoted Myd88 Expression by Competitively Binding to miR-489

**Figure 2:**
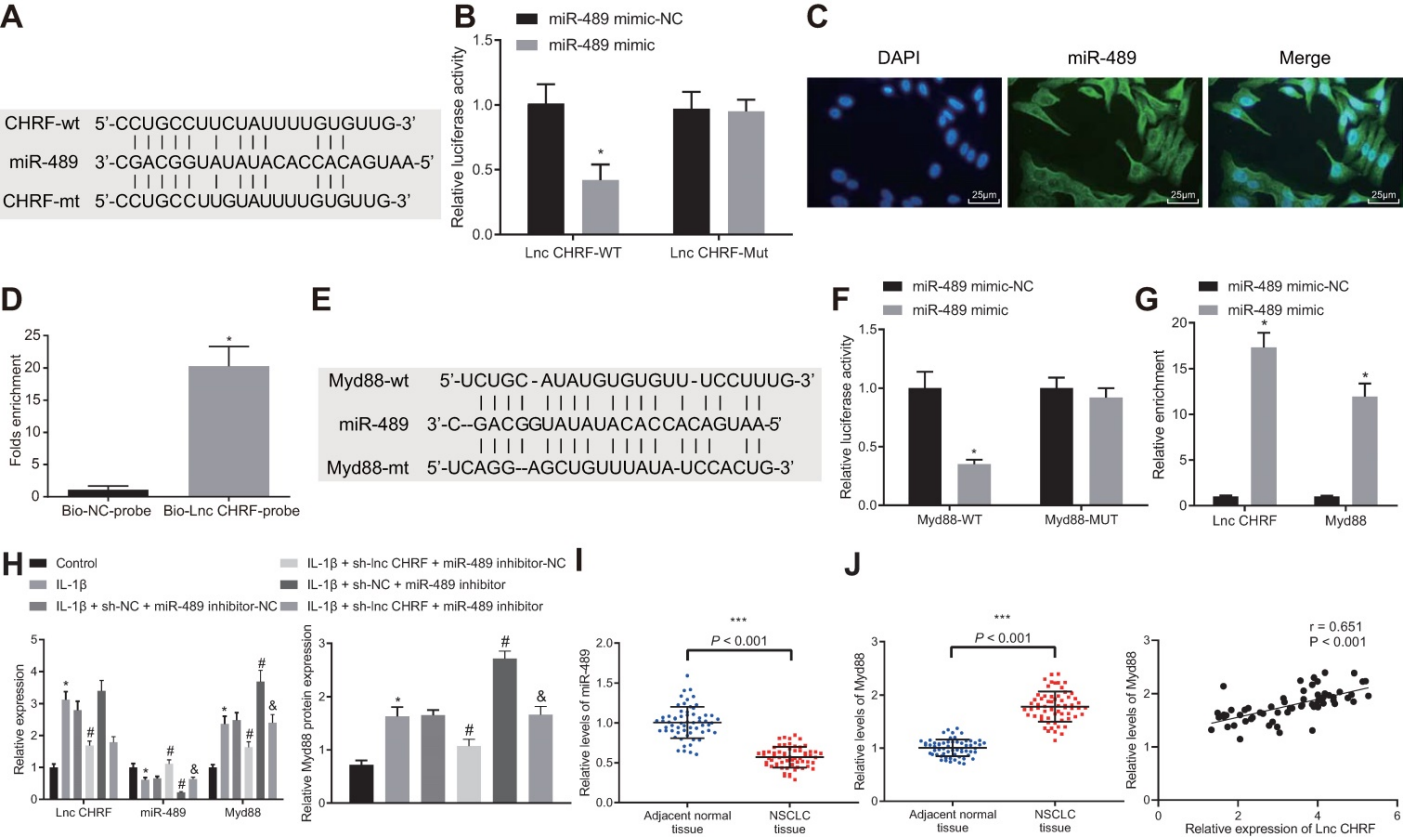
** LncRNA CHRF binds to miR-489 and inhibits miR-489, thereby promoting Myd88 expression. A,** Bioinformatics website was used to predict the targeting relationship between lncRNA CHRF and miR-489. **B,** Dual-luciferase reporter gene assay was used to confirm the targeting relationship between lncRNA CHRF and miR-489 (* *p* < 0.05 *vs.* the miR-489 mimic-NC group). **C,** miR-489 localization in H460 cells through RNA-FISH assay. **D,** RNA pull-down assay was used to verify the direct interaction of lncRNA CHRF targeting miR-489 (* *p* < 0.05 *vs.* the Bio-NC-probe group), scale bar: 25 µm. **E,** Bioinformatics website was used to predict the targeting relationship between miR-489 and Myd88. **F,** Dual-luciferase reporter gene assay was used to further confirm the targeting relationship between miR-489 and Myd88 (* *p* < 0.05 *vs.* the miR-489 mimic-NC group). **G,** RIP assay for interaction between lncRNA CHRF and Myd88 (* *p* < 0.05 *vs.* the miR-489 mimic-NC group). **H,** RT-qPCR was used to detect the expression of lncRNA CHRF, miR-489, and Myd88 in IL-1β-treated cells and Western blot analysis was conducted to detect the protein expression of Myd88 (* *p* < 0.05 *vs.* the control group; # *p* < 0.05 *vs.* the IL-1β + sh-NC + miR-489 inhibitor-NC group; & *p* < 0.05 *vs.* the IL-1β + sh-lncRNA CHRF + miR-489 inhibitor-NC group). **I,** Expression of miR-489 and Myd88 in NSCLC tissues and adjacent normal tissues (n = 62) was detected by RT-qPCR. **J,** Correlation between the expression of lncRNA CHRF and miR-489/Myd88 was analyzed (*** *p* < 0.05 *vs.* the adjacent normal tissues). The experimental values were measurement data and expressed as the mean ± standard deviation. Comparisons between the two groups were analyzed using unpaired *t*-test, while comparisons among multiple groups were assessed using one-way ANOVA, followed by Tukey's post hoc test. The experiment was repeated 3 times independently.

A functional study has suggested that miR-489 is negatively mediated by lncRNA CHRF and can target Myd88, which engages in silica-induced pulmonary fibrosis 8. We focused on the interaction among lncRNA CHRF/miR-489/Myd88 in NSCLC. The bioinformatics website (http://www.targetscan.org/ vert_72/) revealed specific binding site between lncRNA CHRF and miR-489 (Figure 2A), which was validated through luciferase assay (Figure 2B) that the luciferase activity of the WT lncRNA CHRF was reduced when co-transfected with miR-489 mimic, however, no significant change was observed when co-transfected with MUT lncRNA CHRF and miR-489 mimic. Our results from RNA-FISH assays in H460 cells demonstrated that miR-489 was mainly located in the cytoplasm of NSCLC cells (Figure 2C), highly suggestive of a direct interaction between lncRNA CHRF and miR-489. RNA pull-down assay further revealed that miR-489 was enriched in the samples pulled down by the lncRNA CHRF probe compared with the samples pulled down by the NC probe (Figure 2D).

Meanwhile, binding sites between miR-489 and Myd88 were predicted by bioinformatics website (Figure 2E) and validation from luciferase assay exhibited that the luciferase activity of Myd88-WT- Luc was decreased by miR-489 mimic, however, no influence on the Myd88-MUT-Luc (Figure 2F) was observed. Following gain-of function of miR-489 mimic, enrichment of lncRNA CHRF and Myd88 in presence of miR-489 was significantly increased (Figure 2G), indicating that both lncRNA CHRF and Myd88 can bind to miR-489.

To further verify whether lncRNA CHRF can mediate Myd88 expression by binding to miR-489, H460 cells were transfected with sh-lncRNA CHRF and miR-489 inhibitor, followed by treatment of IL-1β. It was evident that the expression of lncRNA CHRF and Myd88 was upregulated while miR-489 expression was downregulated in the cells treated by IL-1β. The delivery of sh-lncRNA CHRF in presence of IL-1β corresponded to downregulation of lncRNA CHRF and Myd88 along with upregulation of miR-489 while delivery of miR-489 inhibitor resulted in downregulation of miR-489 and upregulation of Myd88 yet lncRNA CHRF expression was not significantly different. No significant difference was witnessed regarding lncRNA CHRF expression in H460 cells treated with miR-489 inhibitor in presence of IL-1β + sh-lncRNA CHRF while miR-489 was downregulated and Myd88 was upregulated (Figure 2H).

Furthermore, we found decreased expression of miR-489 in NSCLC tissues and miR-489 showed negative correlation with lncRNA CHRF expression while Myd88 was highly expressed in NSCLC tissues and shared positive correlation with lncRNA CHRF expression (Figure 2I, J).

### LncRNA CHRF-mediated miR-489/Myd88 Axis Promoted Malignant properties of NSCLC Cells after IL-1β Treatment

**Figure 3:**
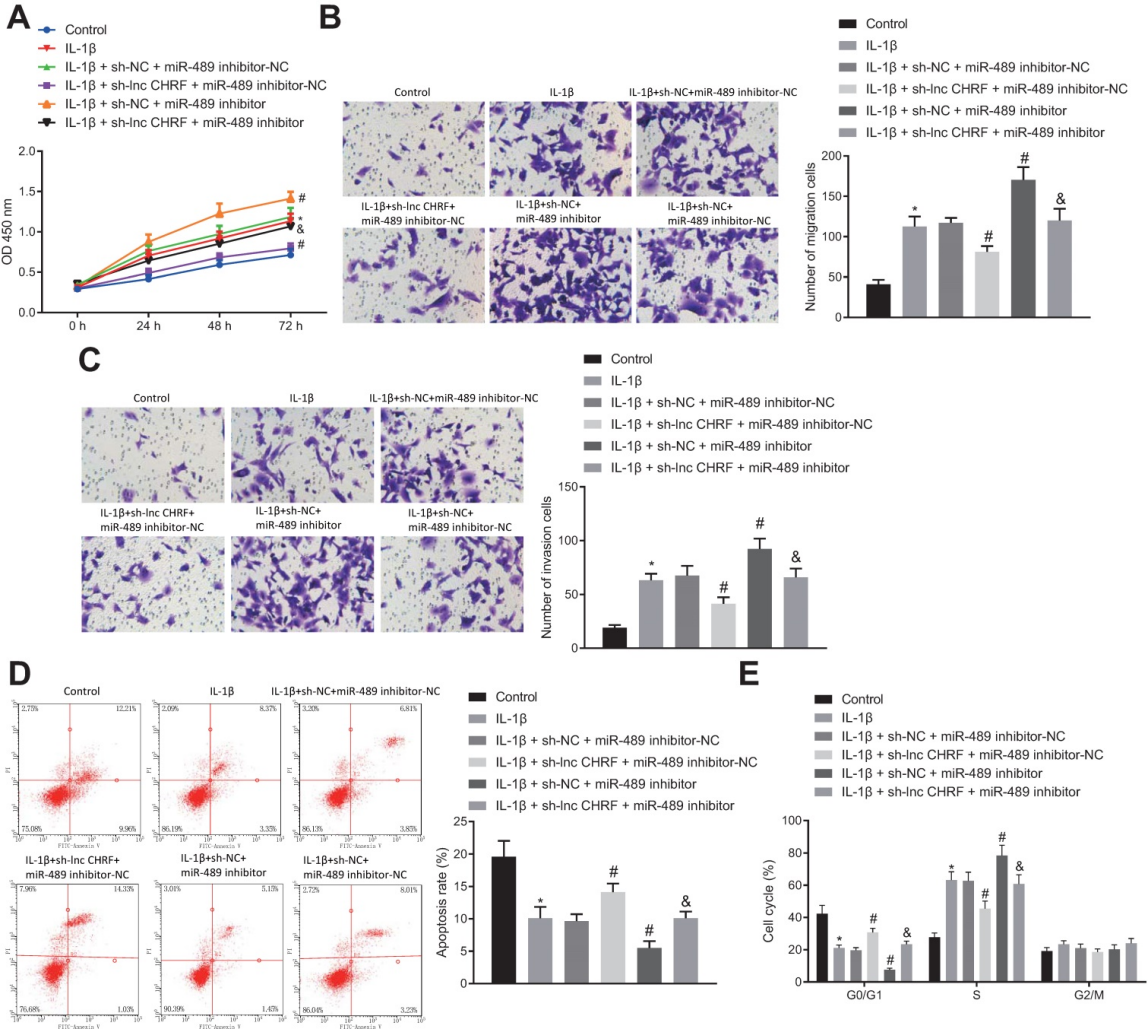
** The lncRNA CHRF/miR-489/Myd88 axis regulates proliferation, apoptosis, migration, and invasion of IL-1β-treated NSCLC cells. A,** Cell proliferation reflected as OD value in each group detected by CCK-8 assay. **B,** Cell migration detected by Transwell assay. **C,** Cell invasion detected by Transwell assay. **D,** Cell apoptosis in each group detected by flow cytometry. **E,** Cell cycle distribution in each group detected by flow cytometry. * *p* < 0.05 *vs.* the control group. # *p* < 0.05 *vs.* the IL-1β + sh-NC + miR-489 inhibitor-NC group. & *p* < 0.05 *vs.* the IL-1β + sh-lncRNA CHRF + miR-489 inhibitor-NC group. The experimental values were measurement data and expressed as the mean ± standard deviation; comparisons among multiple groups were assessed using one-way ANOVA, followed by Tukey's post hoc test. The repeated- measures ANOVA was used to compare the numerical values at different time points, followed by the Bonferroni post hoc test. The experiment was repeated 3 times independently.

The regulatory effects of the lncRNA CHRF/miR-489/Myd88 axis on NSCLC cell functions were analyzed following different transfection protocols and IL-1β treatment in H460 cells. Our result indicated that the cell proliferation, migration and invasion were enhanced by IL-1β treatment while curbed by additional treatment of sh-lncRNA CHRF yet strengthened by miR-489 inhibitor. However, the suppressed cell proliferation, migration and invasion induced by IL-1β + sh-lncRNA CHRF were reversed by further delivery of miR-489 inhibitor (Figure 3A-C).

In addition, flow cytometry for cell apoptosis and cycle distribution showed that IL-1β-induced inhibited cell apoptosis, fewer cells arrested in G0/G1 phase and more cells arrested in S phase were reversed when lncRNA CHRF expression was knocked down and promoted when miR-489 expression was downregulated. Moreover, a decrease in cell apoptosis, reduced number of cells in G0/G1 phase and an increase in the number of cells in S-phase were detected following miR-489 inhibitor treatment in presence of IL-1β + sh-lncRNA CHRF (Figure 3D, E).

### LncRNA CHRF/miR-489/Myd88 Mediated the Tumorigenesis and Metastasis of IL-1β-treated NSCLC Cells *in vivo*

**Figure 4:**
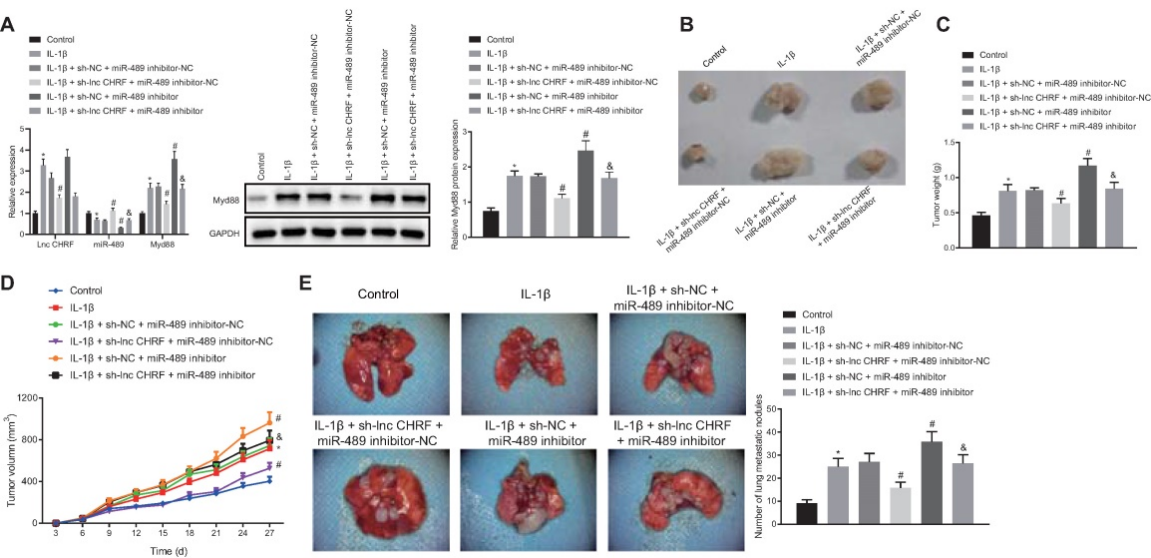
** LncRNA CHRF/miR-489/Myd88 mediates the tumorigenesis and metastasis of NSCLC cells *in vivo*. A,** The expression of lncRNA CHRF, miR-489, and Myd88 was detected by RT-qPCR and Western blot analysis in each group of tumor tissues. **B,** Representative images of tumors from nude mice in each group. **C,** Tumor weight of nude mice in each group. **D,** Tumor volume of nude mice in each group. **E,** The number of nodules in lung tissues of nude mice. * *p* < 0.05 *vs.* the control group. # *p* < 0.05 *vs.* the IL-1β + sh-NC + miR-489 inhibitor-NC group. & *p* < 0.05 *vs.* the IL-1β + sh-lncRNA CHRF + miR-489 inhibitor-NC group. n = 5. The experimental values were measurement data and expressed as the mean ± standard deviation. Comparisons among multiple groups were assessed using one-way ANOVA, followed by Tukey's post hoc test. The repeated-measures ANOVA was used to compare the numerical values at different time points followed by Bonferroni post hoc test.

Following after, H460 cells after transfection were treated with IL-1β and the cell suspension was injected into nude mice to further confirm the effect of lncRNA CHRF on NSCLC *in-vivo*. As displayed by RT-qPCR and Western blot analysis, IL-1β treatment led to upregulation of lncRNA CHRF and Myd88 along with downregulation of miR-489 in tumor tissues. miR-489 inhibitor increased the expression of Myd88, and reduced the expression of miR-489 yet lncRNA CHRF expression did not differ significantly; sh-lncRNA CHRF reduced expression of lncRNA CHRF and Myd88 while elevating miR-489 expression; however, miR-489 inhibitor resulted decreased miR-489 expression and increased Myd88 expression in presence of IL-1β + sh-lncRNA CHRF while no significant difference was witnessed regarding lncRNA CHRF expression (Figure 4A).

Moreover, tumor weight and growth were significantly promoted by IL-1β treatment yet further treatment of sh-lncRNA CHRF resulted in opposite results while further miR-489 inhibitor treatment played a vital catalytic role. In tumor from mice treated with IL-1β + sh-lncRNA CHRF, tumor weight was increased and growth was accelerated by miR-489 inhibitor (Figure 4B-D). Subsequently, IL-1β- treated H460 cells were injected into nude mice via tail vein to further evaluate the lung metastasis. As indicated in Figure 4E, compared with the control group, the number of nodules in lung tissues was significantly increased in presence of IL-1β. When lncRNA CHRF was knocked down, the number of nodules in lung tissues was significantly decreased and opposite changing tendency was observed when miR-489 was knocked down. However, the inhibitory action of sh-lncRNA CHRF on the number of nodules in lung tissues was found to be abolished by miR-489 inhibitor.

## Discussion

**Figure 5:**
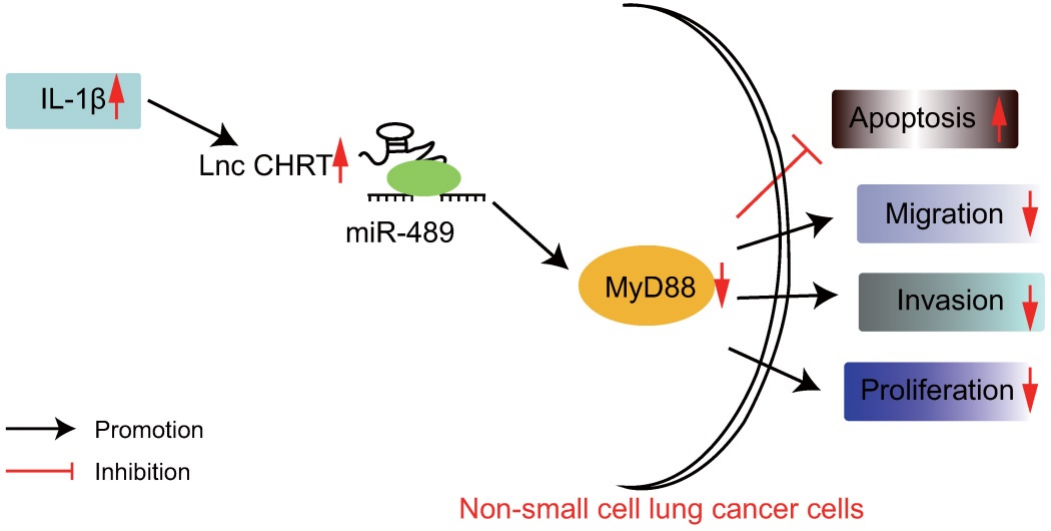
The graphical summary of the function and mechanism of lncRNA CHRF in NSCLC. LncRNA CHRF, upregulated by IL-1β, targets and negatively regulates Myd88 by binding to miR-489, contributes to the progression and development of NSCLC by promoting the NSCLC cell proliferation, migration, and invasion while inhibiting the apoptosis.

Accumulating studies have demonstrated the pivotal roles of lncRNAs in cancer, gene expression control, differentiation, and genomic imprinting and engages in the development and progression of NSCLC *via* functioning as ceRNAs 25, 26. In spite of the fact that numerous lncRNAs are correlated to the metastasis, development, and prognosis of cancers, the effects of lncRNAs on NSCLC remained to be elucidated 27. Recent advancements in oncology have provided miRNAs as novel biomarkers and promising pharmacologic targets in lung carcinogenesis 28. In the present study, we hypothesized that the lncRNA CHRF/miR-489/ Myd88 axis served as novel biomarkers for the treatment and diagnosis of patients with NSCLC and we endeavored to explore the role that IL-1β played in this process. Consequently, this study demonstrated that IL-1β promoted the overexpression of lncRNA CHRF in NSCLC cells. Of note, lncRNA CHRF was found to inhibit the miR-489 expression while indirectly upregulate the expression of Myd88 which results in enhanced malignant properties of NSCLC cells (Figure 5).

We firstly analyzed the IL-1β and lncRNA CHRF expression profile in the cells and tissues of patients with NSCLC to elucidate the potential role of IL-1β and lncRNA CHRF in the pathogenesis of this disease. Our results of ELISA and RT-qPCR analyses showed that IL-1β and lncRNA CHRF were highly expressed in NSCLC cells and tissues. Afterward, IL-1β induced elevation of lncRNA CHRF in NSCLC cells and tissues. Consistently, IL-1β possesses promoting effect on the mitogen-activated protein kinase-dependent invasion along with epithelial to mesenchymal transition (EMT) via Matrigel™, thus, depicting its role as a pro-invasive factor in NSCLC 29. Further studies have found that high expression of the serum IL-1 receptor antagonist, leads to the poor prognosis of patients with NSCLC, reflecting its prognostic role in this disease 30. Nevertheless, a pro-inflammatory cytokine of IL-1β functions as a key regulator in the extracellular matrix production as well as the proliferation of human fibroblasts from lung 31. Particularly, the level of IL-1β in serum has been revealed to effectively predict the prognosis of patients suffering from NSCLC 32. More importantly, downregulation of L-1β has been attributed to the decreased incidence and fatality in lung cancer 33. Collectively, these above-described findings reflect the key role of IL-1β in NSCLC.

Notably, our follow-up experiments revealed the interaction among lncRNA CHRF, miR-489, and Myd88. Moreover, we also reported the role of lncRNA CHRF as a ceRNA, exhibiting the ability to bind to and inhibit the miR-489 expression, and results in indirect upregulation of the Myd88 expression, which was negatively regulated by miR-489. Furthermore, the RT-qPCR results exhibited an upregulation in lncRNA CHRF and Myd88 expression whereas a downregulation in miR-489 expression after transfection of the miR-489 inhibitor, which collectively suggested that lncRNA CHRF effectively inhibited the miR-489 expression while enhancing the Myd88 expression. Consistently, Wang *et al.* indicated lncRNA CHRF acted as an endogenous 'sponge' of miR-489 participating in the downregulation of miR-489 expression and upregulation of Myd88 expression in hypertrophy 12. According to a previously reported study, lncRNA CHRF significantly promotes cardiac hypertrophy via the Akt3/miR-93 axis 34. Xie *et al.* have further verified that downregulated miR-489 enhances the invasion of NSCLC cells by targeting SUZ12, a polycomb protein correlated with human cancer pathogenesis 35, 36. Additionally, miR-489 shares inverse correlation with lncRNA CHRF in colorectal cancer (CRC) tissues whereas the downregulation of miR-489 by lncRNA CHRF overexpression promotes the metastasis and EMT process of CRC cells through the TWIST1/EMT axis, which is partially consistent to our experimental results 37. Collectively, these above-reported findings signify the vital role of lncRNA CHRF/miR-489/Myd88 axis in NSCLC. Finally, we aimed to further reveal the detailed role of the lncRNA CHRF/miR-489/Myd88 axis in NSCLC. In consent with above-reported findings, our data exhibited suppressed malignant properties of NSCLC cells after transfection of sh-lncRNA CHRF while miR-489 inhibitor reversed these above-described effects, thus, indicating that downregulation of lncRNA CHRF suppressed malignant properties of NSCLC cells.

## Conclusion

In summary, our data exhibited the high expression of IL-1β in the NSCLC cells and tissues, which further promotes the overexpression of lncRNA CHRF. Moreover, lncRNA CHRF inhibits the miR-489 expression but elevates the Myd88 expression, thus promoting the malignant properties of NSCLC cells. Therefore, we suggest that lncRNA CHRF/miR-489/Myd88 axis serves as a potential therapeutic target for the intervention and treatment of NSCLC. Nevertheless, further researches on the efficacy of these biomarkers in the intervention and treatment of NSCLC on clinical trials should be focused in the future.

## Supplementary Material

Supplementary tables.

## Abbreviations

LncRNAs: Long noncoding RNAs
NSCLC: non-small-cell lung cancer
CHRF: cardiac hypertrophy-related factor
miR-489: microRNA-489
Myd88: myeloid differentiation factor 88
IL-1β: interleukin-1β
RPMI: Royal Park Memorial Institute
ELISA: Enzyme-linked immunosorbent assay
RT-qPCR: Reverse transcription-quantitative polymerase chain reaction
NC: negative control
GAPDH: Glyceraldehyde-3-phosphate dehydrogenase
FISH: RNA Fluorescence *In situ* Hybridization
DEPC: diethylpyrocarbonate
PBS: phosphate-buffered saline
WT: wild type
MUT: mutant
Ago2: Argonaute 2
IgG: immunoglobulin G
PI: propidium iodide
SPSS: Statistical Package for the Social Sciences
ANOVA: analysis of variance
ceRNA: competitive endogenous RNA
EMT: epithelial to mesenchymal transition
CRC: colorectal cancer
