# Supplementary material for: IL-1β-Triggered Long Non-coding RNA CHRF Induces Non-Small Cell Lung Cancer by Modulating the microRNA-489/Myd88 Axis
Source: J Cancer. 2022 May 16;13(8):2620–30. doi: 10.7150/jca.63256 (PMC9174862; doi:10.7150/jca.63256)
Supplement: Supplementary file 1 — Supplementary tables. [file jcav13p2620s1.pdf]

Supplementary Table 1 | Clinicopathological characteristics of patients with NSCLC

Characteristics		N
Gender		
	Male	38
	Female	24
Age (years)		
	≤ 40	21
	> 40	41
T stage		
	I	35
	II - III	27
N stage		
	I	34
	II - III	28
TNM stage		
	I	29
	II - III	33

Note: NSCLC, non-small-cell lung cancer; TNM, tumor node metastasis.

Supplementary Table 2 | RT-qPCR primer sequences

Genes	Sequences (5' - 3')
LncRNA CHRF	F: CAACTTTACCCATCTCTTCTC
	R: CTGAATTACTTCAGAGGAAAG
miR-489	F: GCAGCTTGGTGGTCGTATGTG
	R: Universal reverse primer
Myd88	F: CGCCGGATGGTGGTGGTTGT
	R: TGTAGTCGCAGACAGTGATGAACC
U6	F: AAAGCAAATCATCGGACGACC
	R: Universal reverse primer
GAPDH	F: GGAGCGAGATCCCTCCAAAAT
	R: GGCTGTTGTCATACTTCTCATGG

Note: RT-qPCR, reverse transcription quantitative polymerase chain reaction; LncRNA CHRF, long noncoding RNA cardiac hypertrophy-related factor; miR-489, microRNA 489; Myd88, myeloid differentiation factor 88; GAPDH, glyceraldehyde-3-phosphate dehydrogenase; F, forward; R, reverse.
